# Differentiating between normal and inflammatory blood serum samples using spectrochemical analytical techniques and chemometrics

**DOI:** 10.1007/s00216-025-05802-6

**Published:** 2025-03-06

**Authors:** Rania M. Abdelazeem, Zienab Abdel-Salam, Mohamed Abdel-Harith

**Affiliations:** 1 Engineering Applications of Laser Department, National Institute of Laser Enhanced Science, Cairo University, Giza, 12613 Egypt; 2 Laser Applications in Metrology, Photochemistry and Agriculture Department, National Institute of Laser Enhanced Science, Cairo University, Giza, 12613 Egypt

**Keywords:** Laser-induced breakdown spectroscopy, Laser-induced fluorescence, Inflammation, Blood serum, PCA, Graph theory

## Abstract

Inflammation detection in blood serum samples is commonly performed using clinical analyzers, which are expensive and complex and require specific labels or markers. Spectrochemical analytical techniques, such as laser-induced breakdown spectroscopy (LIBS) and laser-induced fluorescence (LIF), have emerged as alternative methods for qualitative and non-destructive analysis in various fields. This study explores applying LIBS and LIF techniques for label-free discrimination between normal and inflammatory blood serum samples. In the LIBS analysis, the serum samples are deposited on ashless filter paper and exposed to a high-power Nd:YAG laser source to induce plasma emission. The emitted light is dispersed in a spectrometer and an ICCD camera that captures the spectral lines. The LIF technique utilizes a diode-pumped solid-state laser source to excite the blood serum sample placed in a quartz cuvette. The resulting emission spectra are collected and analyzed using a spectrometer equipped with a CCD detector. The obtained spectroscopic data from both techniques is subjected to principal component analysis (PCA) and graph theory for classification and clustering. The PCA classified the two classes with a data variance of 85.4% and 92.8% based on the first two principal components (PCs) for LIBS and LIF spectra. The graph theory clustered the two classes with an accuracy of 76% and 100% based on LIBS and LIF spectra. The statistical methods effectively discriminate between normal and inflammatory serum samples, providing satisfactory results. The proposed spectrochemical methods offer several advantages over traditional clinical analyzers. They are cost-effective and rapid, making them suitable for the fast and reliable identification of serum samples in laboratories. The non-destructive nature of these techniques eliminates the need for specific labels or markers, further streamlining the analysis process.

**Graphical Abstract:**

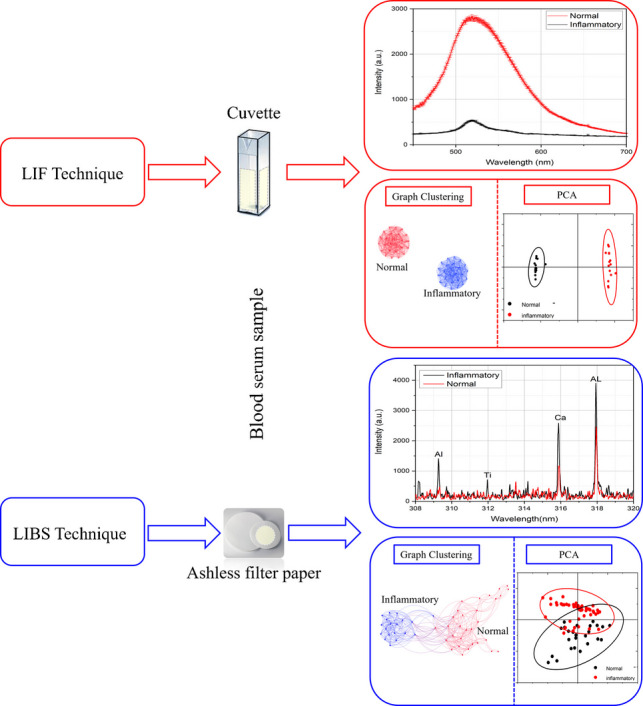

## Introduction

Clinical diagnoses are frequently performed based on blood samples [1–3]. Blood serum is a liquid portion of the blood without clotting factors and cells. Thus, it contains proteins and other molecules representing the whole-body system [4]. Acute inflammation or infection is related to a specific protein type called C-reactive protein (CRP). CRP deviates by at least 25% during inflammatory disorders. It has been found that the maximum concentration of CRP associated with acute bacterial infections increased up to 1000-fold [5]. CRP is synthesized in organs, such as liver hepatocytes, smooth muscle cells, macrophages, endothelial cells, lymphocytes, and adipocytes [6]. The levels of CRP in plasma increase from around 1 µg/mL to over 500 µg/mL within 24–72 h of severe tissue damage such as cancer and trauma [7]. Several factors impact CRP levels, such as gender, age, weight, blood pressure, smoking status, and lipid levels [8]. It has been found that the average CRP level in a healthy Caucasian is about 0*.*8 mg/L. However, this baseline can change due to many other factors, including polymorphisms in the CRP gene [9].

Inflammation detection is performed in laboratories via clinical analyzers. However, they are expensive to purchase, operate, and maintain. Besides, in some cases, they need specific labels or markers that may destroy the sample. Additionally, they are complex devices that require particular training to operate effectively. Therefore, spectrochemical analytical techniques can be utilized as uncomplicated, rapid, cost-effective, non-destructive, and label-free means to monitor and diagnose biological sample disorders. These techniques involve laser-induced breakdown spectroscopy (LIBS) and laser-induced fluorescence (LIF).

LIBS is a multi-element analytical technique [10] widely used in various fields, such as forensics, metallurgy, pharmaceuticals [11], and biology [12, 13]. The great benefit of this technology is its ability to provide quantitative and qualitative analysis of trace elements with no or minimal sample preparation [14–16]. It is one of the atomic emission spectroscopic procedures based on laser ablation utilizing a high-power laser source [13]. LIBS is generally based on the Nd:YAG laser because of its compactness and highly focused high-energy pulses. The excitation laser generates plasma from the sample. The atoms in the produced plasma provide distinctive spectral lines that the spectrometer detects when they return to lower energy levels. The emitted spectral lines of the plasma have been analyzed to determine the sample’s elemental composition. For biomedical applications, LIBS has been widely used in the chemical analysis and classification of hard tissues such as teeth or bones [17] and for soft tissues as well [18]. Additionally, LIBS has become widely used in the spatially resolved analysis of basic metals such as Zn, Cu, Fe, and non-metals in biological tissues [19]. Besides, blood samples have been analyzed using LIBS by different methods, e.g., deposition on a glass slide, filter paper [20], dry droplet preparation, freezing [21], or extraction by paraffin wax [22]. The authors of [23] demonstrated that integrating LIBS with a multiplicative scatter correction-mean impact value-backpropagation neural network (MSC–MIV–BPNN) model can distinguish between inflammatory and healthy blood serum samples. However, the complexity of the proposed neural network model presents challenges in interpreting the results. Moreover, the training phase of the model is crucial and necessitates large datasets for effective generalization. On the other hand, the LIBS spectra they presented in this study suffer from low signal-to-noise ratios (SNR), which can impact the accuracy of spectral analysis and potentially lead to misinterpretations. It is worth mentioning that LIBS can be used as a non-destructive tool for inflammation detection without specific markers/labels or sample preparation [24]. In addition, it offers near-instantaneous analysis results, making it suitable for time-sensitive applications. It also has a low cost compared to commonly used clinical analyzers and limits personal intervention, which causes significant errors [23].

Laser-induced fluorescence (LIF) is a non-destructive visualization technique that can instantaneously detect the fluorescence of existing fluorescent molecules to show their concentration, density, temperature, velocity, and even pressure [25]. This technology has several benefits, such as sensitivity and selectivity [26]. Sensitivity is because the intensity of the fluorescence signal is proportional to the intensity of the excitation light. In addition, the ability to separate the emission and excitation signals from the background. Recently, various laser sources and optical fibers have been developed to transmit and collect excitation and fluorescence signals from the human body from the external environment. Moreover, biomedical research has exploited LIF spectroscopy to analyze biological samples, including blood serum, to identify changes associated with various conditions, including inflammation, cancer detection [27, 28], and DNA genetic analysis [29, 30]. Therefore, this technique proved its effectiveness in tissue diagnosis and blood analysis. In this technique, a laser beam is used to excite a sample under test, and then the emitted fluorescence is collected and fed to a suitable detector for display. The emitted fluorescence is considered a fingerprint for specific molecules in the sample. The LIF technique can be used more than traditional clinical analyzers as a supreme non-destructive technique. It is cost-effective due to limited reagent use and rapid analysis. Besides, it can be designed for portable use, enabling bedside testing. Furthermore, it facilitates the direct analysis of biological samples [31], reducing the risk of contamination and the preparation time. Unlike clinical analyzers that cause increased generation of noise, heat, and vibrations [32], the LIF technique does not produce any of these effects and the personal intervention in the LIF technique is quite minimal.

In spectrochemical analysis, it is common to use two techniques, one for elemental analysis and the other for molecular analysis, to validate each other’s findings. For example, the authors of references [33, 34] employed LIBS and LIF, while the authors of reference [35] utilized LIBS and Raman spectroscopy.

This study proposes straightforward techniques that avoid data manipulation, utilizing LIBS, LIF, and chemometrics to differentiate between normal and inflammatory blood serum samples. A total of 50 specimens were analyzed, consisting of 25 normal and 25 inflammatory samples. In the LIBS technique, blood serum samples were deposited on high-quality ashless filter paper and allowed to dry for 15 min in a clean and dry laboratory environment. A high-power Nd:YAG laser was used as the ablation source. The light emitted from the laser-induced plasma was then dispersed and detected using a spectrometer equipped with an ICCD detector. In the LIF technique, a diode-pumped solid-state laser (DPSS) at *λ* = 405 nm was used to excite the blood serum samples. The emitted fluorescence light was collected using a spectrometer.

The obtained spectra from both techniques have been classified and clustered using the principal component analysis (PCA) and the graph theory approaches.

## Materials and methods

### Blood serum sample preparation

The blood serum samples employed in this framework were prepared at Al-Kasr Al-Aini Hospital, Cairo University. The investigated samples’ protocol has been reviewed by the “NILES’ Ethics Committee at Cairo University,” which approved it. Blood samples were converted into serum by following some basic steps. The blood sample was collected into a microcentrifuge tube, allowing it to clot by leaving it undisturbed at room temperature for 15–30 min. Then, the clot was removed by centrifuging for 10 min in a refrigerated centrifuge. Following the centrifugation, the serum was immediately transferred into a clean microcentrifuge tube using a pipette. The collected samples should be stored at 2–8°C while being handled. In case of storage for further analysis, it should be stored at −20°C or lower. It is worth mentioning that multiple serum freezes should be avoided because this is detrimental to many serum components.

### Laser-induced breakdown spectroscopy measurements

**Fig. 1:**
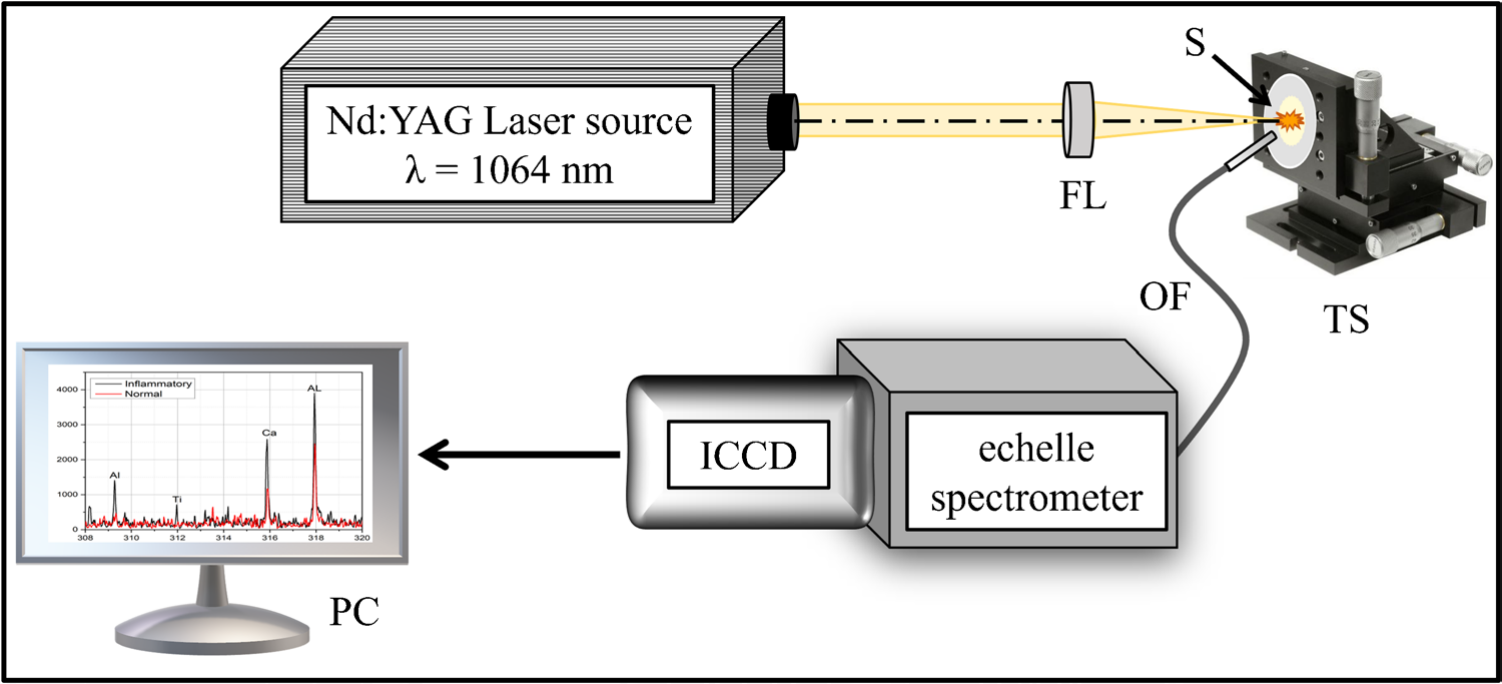
Schematic layout of the laser-induced breakdown spectroscopy (LIBS) experimental setup. FL, focusing plano-convex lens with a focal length of 100 mm; S, blood serum sample onto ashless filter paper; TS, translation stage; OF, optical fiber

The experimental configuration of the LIBS instrumentation has been described previously in [34]. A standard LIBS setup, demonstrated in Fig. 1, has been used in the current work, in which a Q-switched Nd:YAG laser (Brio, Quantel, France) produces laser pulses of 50 mJ/pulse at a wavelength *λ* = 1064 nm with a pulse width of 5 ns and a repetition rate of 20 Hz. The serum samples (0.5 mL per droplet) were placed onto ashless filter paper (Cat. No. 1444-090, thickness 176 µm, Whatman, Maidstone, UK). They were allowed to sit aside in clean, dry air for approximately 15 min to ensure that the serum had been evenly distributed and absorbed by the filter paper, preparing it for analysis via LIBS. The laser pulses have been focused on the air under atmospheric pressure onto the sample’s surface using a plano-convex quartz lens (FL) with a focal length of 10 cm. An x-y micro-metric translational stage (TS) was used to control the sample holder’s position in front of the focused laser beam. The laser pulses were delivered as a single pulse ten times adjacent and in succession, each pair spaced 20 µm apart. This process was repeated on the sample surface in five locations to collect 50 spectra. The filter paper was stretched on a flat substrate; the laser beam did not penetrate the filter paper and reach the substrate material.

A 2-m-long fused silica optical fiber (OF) having a 600-µm core diameter was used to collect the light emitted from the laser-induced plasma. The collected light is fed to the entrance slit of an echelle spectrometer (Mechelle 7500, Multichannel Instruments, Sweden) coupled to an intensified charge-coupled device (ICCD camera) as a detector (DiCAM-Pro, PCO, Computer Optics, Germany). The ICCD has been triggered optically to exclude any probable electronic interference and jitters. The spectra have been collected at an optimized delay time of 1500 ns and gate width of 2500 ns to minimize the continuum emission. As mentioned above, 50 spectra were collected from 10 laser shots on five new spots separated by 1 mm on the same blood serum sample, then averaged to compensate for any surface inhomogeneity. Emission spectra treatment and identification of the spectral lines were performed using the commercial software LIBS++ [36].

### Laser-induced fluorescence measurements

**Fig. 2:**
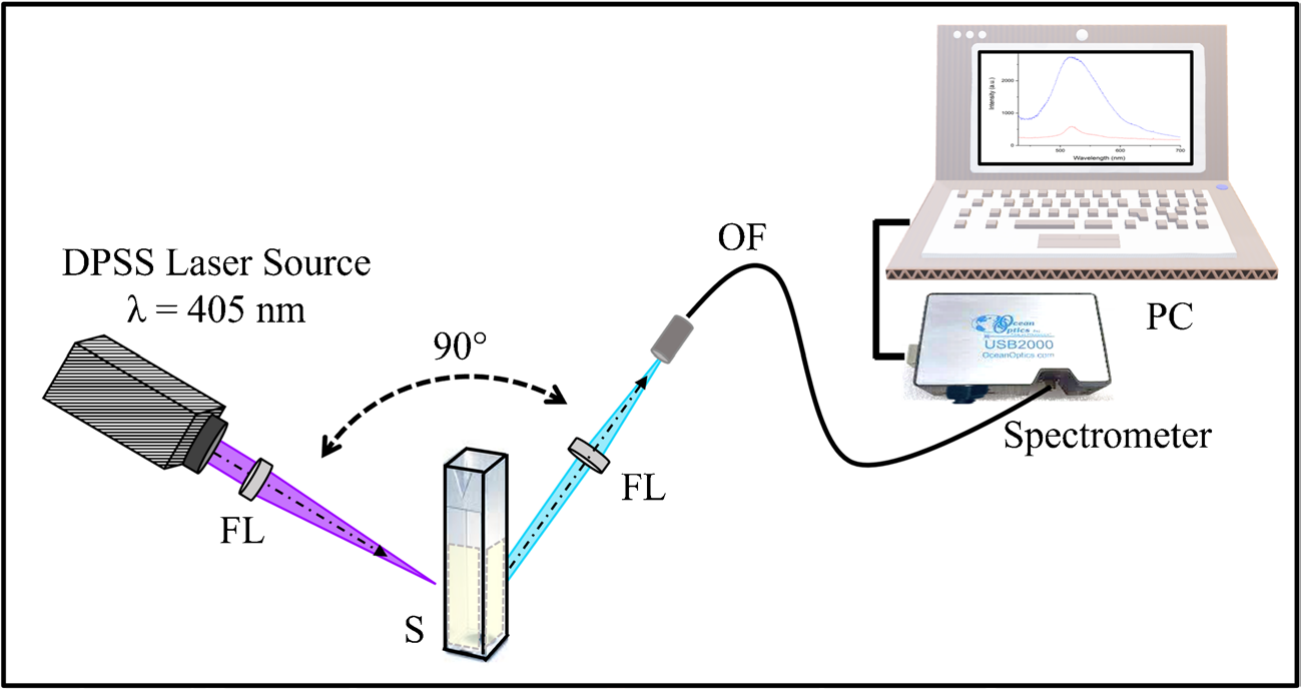
Schematic layout of the laser-induced fluorescence (LIF) experimental setup. DPSS, diode-pumped solid-state laser source with a wavelength *λ* = 405 nm; OF, optical fiber; FL, focusing plano-convex lens with a focal length of 50 mm; S, blood serum sample

A DPSS (Changchun New Industries Optoelectronics Tech Co, Ltd, China) with a wavelength of 405 nm and an average power of 20 mW was used to excite the blood serum samples, as demonstrated in Fig. 2. The blood serum sample was placed in a spectroscopic quartz cuvette (1-cm path length). The emitted fluorescence signal has been collected perpendicularly using an optical fiber equipped with a collimator. The emitted fluorescence signal has been fed into a compact spectrometer (USB2000 FLG, Ocean Optics, USA). The fluorescence spectrum is the average of thirty collected spectra for each sample to compensate for possible scattering effects and instrumental electronic drift that may artificially affect the measurement accuracy. The spectra obtained from the spectroscopic system are acquired and analyzed using the commercial SpectraSuite software (Ocean Optics, USA) for dispersion and detection.

### Principal component analysis

The PCA is a statistical multivariate analytical method that efficiently reduces the dimensionality of spectra to extract the most crucial spectral feature variables by correlating the input data [37]. It has been widely used to analyze exploratory data, pattern recognition, and classification [13]. The PCA technique employs three significant outputs: (i) **variance**, (ii) **loadings**, and (iii) **scores**. The data points are distributed in *K*-dimensional space, where *K* is the number of obtained PCs, and the dataset will be described by a parameter called variance. The **variance** is defined as the sum of standard deviations from the sample mean divided by the total number of samples minus one. The obtained PCs are arranged in descending order with respect to the variance. Therefore, only a few PCs will carry significant information to describe the original dataset. The total variance explained by PCs should ideally be high, typically above 70% [38]. The remaining variance represents information in the original data not captured by the PCA model, which may be less relevant data for classification or noise. The **loadings** describe the relation between the newly obtained PCs and the original dataset. The **scores** are the coordinates of the original objects in the newly constructed low-dimensional space. It is worth mentioning that the broader distribution of the points in the cluster obtained by the PCA model means higher data fluctuations.

In this study, the measured data were analyzed and classified using PCA through commercial software (Origin Pro 2019b) to obtain new variables known as principal components (PCs), which are linear combinations of the original variables. PCA was used to analyze the variations in the spectral data from normal and inflammatory blood serum samples acquired via LIBS and LIF.

### Data analysis using graph theory

The acquired spectra (each sample is represented by one spectrum) from LIBS and LIF are analyzed using an unassisted classification approach called the graph theory. This approach has been recently used in various fields such as Genetic Genealogy [39], social networks analysis [40], forensic applications [41], and spectroscopy [42]. It is based on estimating one correlation matrix for the LIF spectra and another for the LIBS spectra. The correlation matrix is interpreted as an adjacency matrix representing the links between the different spectra. Each spectrum is represented as one vertex of the network, while the correlation between each two spectra is expressed by a link or edge that connects two vertexes. The expression of the network by vertexes and links yields an undirected network (i.e., the link strength between the two vertexes, *A* and *B*, is the same as between *B* and *A*). It should be noted that the spectra of different groups generally have a non-zero correlation. The correlation matrix between the two spectra, *A* and *B*, is expressed as [43]:

1 $${C}_{A,B}=\frac{\sum_{i=1}^{n}{A}_{i}{B}_{i}}{\sqrt{({\sum }_{i=1}^{n}{A}_{i}^{2})({\sum }_{i=1}^{n}{B}_{i}^{2})}}$$

where *n* is the number of spectral points that express the measured intensity values. The correlation matrix (*C*) is symmetric and has dimensions *m × m*, where *m* is the number of samples involved in the classification process, i.e., in our case, the dimensions of the matrix *C* are 50 *×* 50 as we have 50 samples. By applying Eq. 1, the value of each element of the matrix *C* ranges between 0 and 1. This approach provides a statistical tool called the “modularity parameter” for the discrimination between the different groups based on their edge weights; therefore, the group with a strong correlation can be separated from the one that results from a generic similarity between the spectra. This parameter is maximized by applying a threshold value to the edge values. Therefore, the graph can be divided into classes or clusters whose members have strong connections between them and weak connections with the other members. After estimating the maximum modularity parameter, the different spectra could be automatically classified into clusters.

It is worth mentioning that the graph theory approach has several advantages compared to the traditional chemometric approaches that are commonly used along with the spectrochemical analytical methods such as PCA and partial least squares regression (PLSR). In contrast to the PCA method, which reduces the data’s dimensionality, the graph theory approach preserves the structure of the original spectra. Besides, it can easily detect the non-linear relationship between the spectra. Furthermore, it provides intuitive visualizations of complex spectral relationships, facilitating easier interpretation of the obtained results [41].

## Results and discussion

### Laboratory results

The collected samples were from females aged 45 to 47. They were analyzed in the laboratory using the fully automated clinical analyzer model BiOLiS 50i to obtain CRP and total protein for inflammation detection.

The laboratory analysis of CRP for normal samples showed an average value of 2.18 mg/dL with a standard deviation of approximately 1.58 mg/dL and an average value of 126.4 mg/dL with a standard deviation of roughly 10.46 mg/dL for the inflammatory samples. At the same time, the average value of total protein (TP) was 7.28 mg/dL for both normal and inflammatory blood serum samples.

### LIBS results

**Fig. 3:**
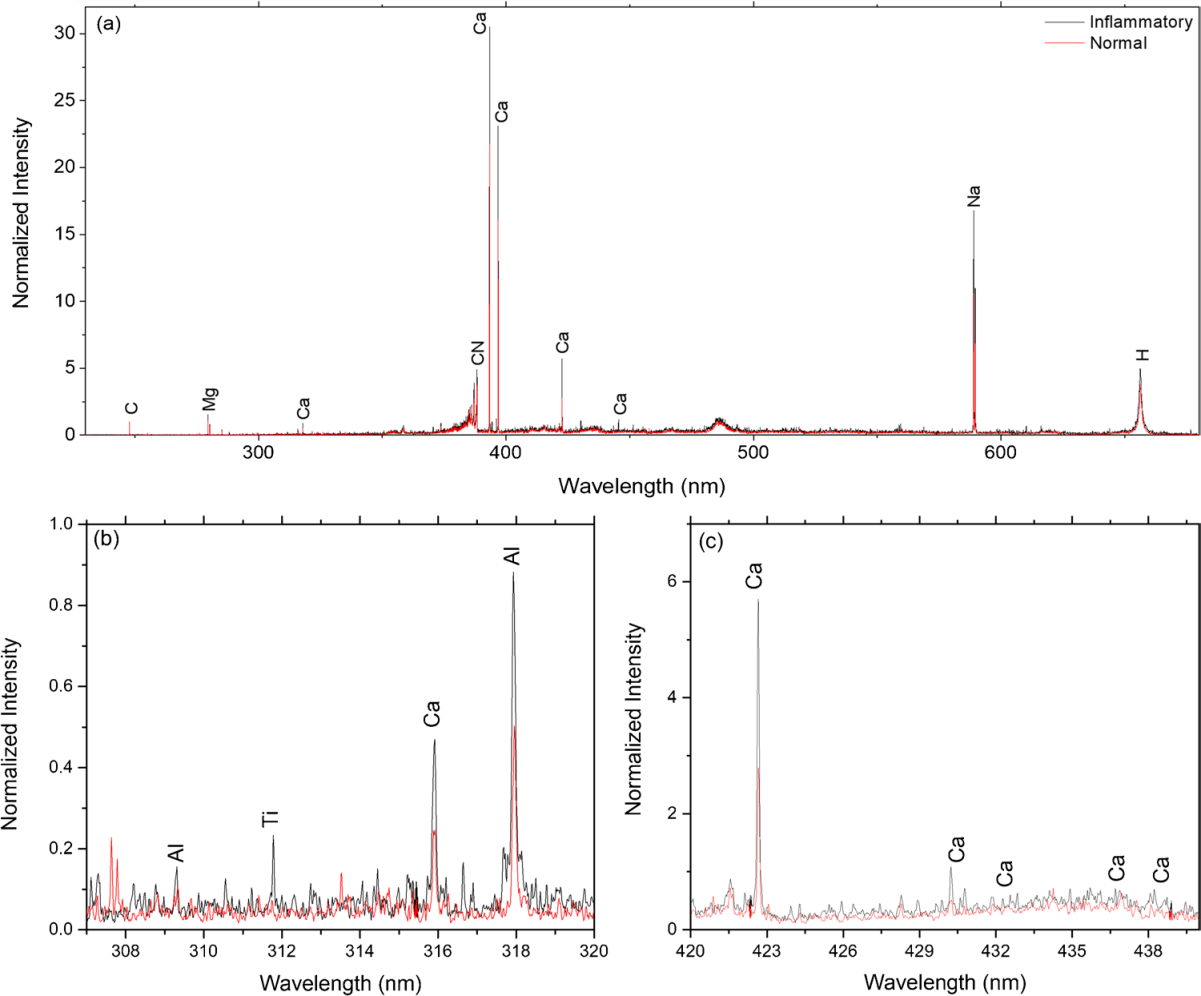
**a** The obtained LIBS spectra of the normal and inflammatory blood serum samples, **b**, **c** the zoomed parts of the spectrum showing spectral lines of elements of interest. All spectra are normalized to the carbon atomic line at 247.8 nm

Figure 3 a shows the obtained LIBS spectra. The results show that the inflammatory blood serum samples exhibit higher levels of aluminum (Al), titanium (Ti), and calcium (Ca) elements in the wavelength range of 308 to 440 nm, as shown in Fig. 3 b and c.

The higher levels of Al and Ti are not related to inflammation. As depicted in the LIBS spectra, those elements in the inflammatory serum samples could be related to certain medications or medical treatments [44, 45]. The interpretation of these elements should be performed in conjunction with other biochemical, clinical, and diagnostic parameters to understand the potential implications and underlying mechanisms contributing to the presence of these elements in the inflammatory serum samples.

Higher levels of Ca in the blood serum (hypercalcemia) are usually associated with inflammatory conditions. Inflammatory processes can sometimes lead to the release of Ca from bone tissue into the blood serum. Moreover, certain inflammatory conditions or diseases may affect the regulation of Ca in the body, leading to disturbances in Ca metabolism. On the other hand, Ca ions themselves can also play a role in inflammation. Ca ions are involved in various cellular signaling pathways, including those related to inflammation and immune responses. Changes in intracellular Ca levels can influence immune cells’ activation and function. The secretion of cytokines from cells is also a fundamental response to infection and inflammation in the body.

### LIF results

**Fig. 4:**
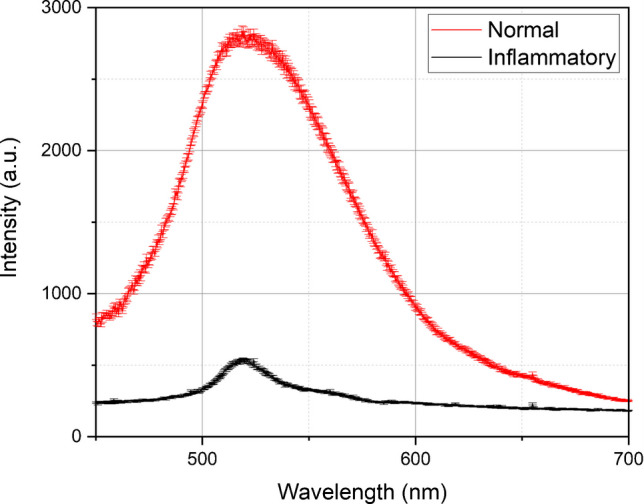
The obtained LIF spectra of the normal and inflammatory classes. Note that for each spectrum, each data point on the plot represents the mean of 25 individual intensity measurements, and the error bars correspond to the standard deviation calculated for each set of 25 measurements

Fifty blood serum samples, 25 normal and 25 inflammatory, have been tested using the LIF experimental setup. The samples were excited at 405 nm, which induces a fluorescence emission spectrum with a maximum emission peak at 518 nm. The average of the obtained LIF spectra with the standard deviation of 25 normal and 25 inflammatory samples is depicted in Fig. 4. It should be noted that the LIF spectra were unnormalized because we were interested in absolute intensity values for quantitative comparison between the two classes. At the same time, the experimental conditions and instrumental parameters are consistent across all measurements.

The results reveal that the fluorescence intensity of the normal blood serum sample is 5.3 times higher than that of the inflammatory samples. The discrimination peak between the two groups has been found at a wavelength of 518 nm. It can be concluded that the higher peak of the normal samples compared to the inflammatory samples could be related to the presence or concentration of certain fluorescent compounds or biomolecules between the two samples. Inflammation can alter the levels of specific molecules that exhibit fluorescence at 518 nm. For instance, it can cause a decrease in the concentration of particular fluorophores like advanced glycation end-products (AGEs) or other fluorescent metabolites, which might result in a lower emission peak for inflammatory samples. AGEs interact with specific receptors (RAGEs—receptors for AGEs), triggering cellular responses that promote inflammation and oxidative stress. Certain endogenous fluorophores in serum might exhibit changes in their fluorescence properties in inflammatory conditions. These changes could be due to alterations in the molecular environment or the presence of specific biomolecules associated with inflammation.

### Classification results based on PCA

#### PCA of LIBS spectra

**Fig. 5:**
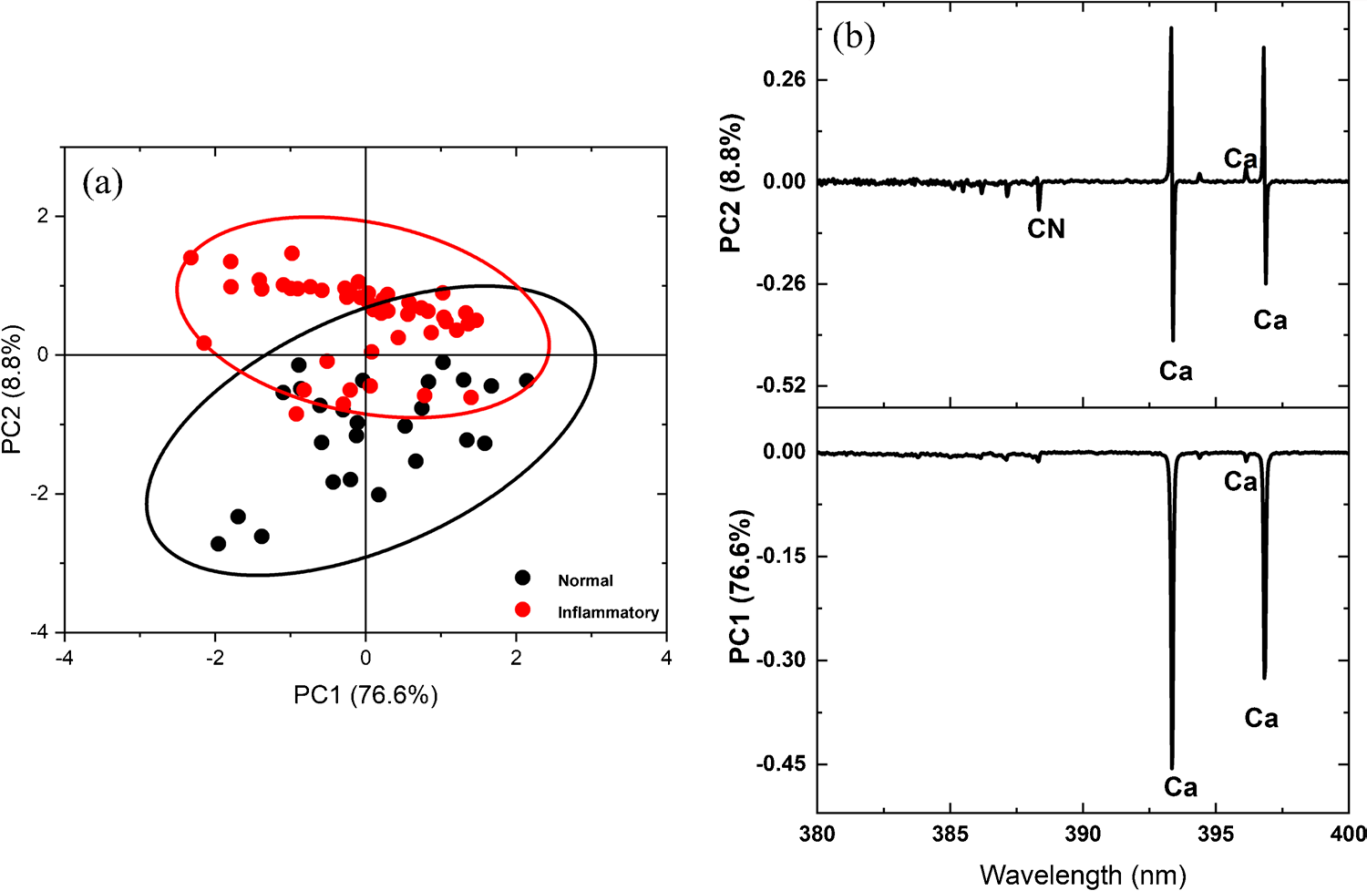
**a** Score plot of PC1 vs PC2 of a principal component analysis on LIBS spectra of normal and inflammatory serum samples. **b** PCA loadings plots of PC1 and PC2

PCA was utilized as a multivariate, unsupervised statistical method to differentiate between normal and inflammatory blood serum samples. Twenty-five spectra from each sample type were analyzed, covering the entire range of each spectrum (200–750 nm). The results, depicted in Fig. 5 a, indicate that only two principal components are required to distinguish between normal and inflammatory samples. It is evident that the data from normal blood serum samples clustered on the negative side of PC2, while the data from inflammatory blood serum samples clustered on the positive side of PC2 on the plot. PC1 and PC2 together represent 85.4% of the data variance, with PC1 accounting for 76.7% and PC2 for 8.8%. Therefore, the PCA demonstrates a clear spectroscopic difference between normal and inflammatory blood serum samples.

Figure 5b depicts the loadings plots in the 380–400 nm spectral range. The upper graph shows the spectral features of Ca and CN that may be produced from the cytokines proteins that help control inflammation in the body are in the negative sector of PC2, indicating normal blood serum, as shown in Fig. 5 a. Furthermore, Ca lines also appear in the negative sector of PC2, affirming the corresponding score plot of the inflammatory blood serum. The obtained results reveal that PCA loading plots of the LIBS spectra clarify the calcium lines’ dominance as a discriminatory element of blood inflammation with minimal influence of the noise.

#### PCA of LIF spectra

**Fig. 6:**
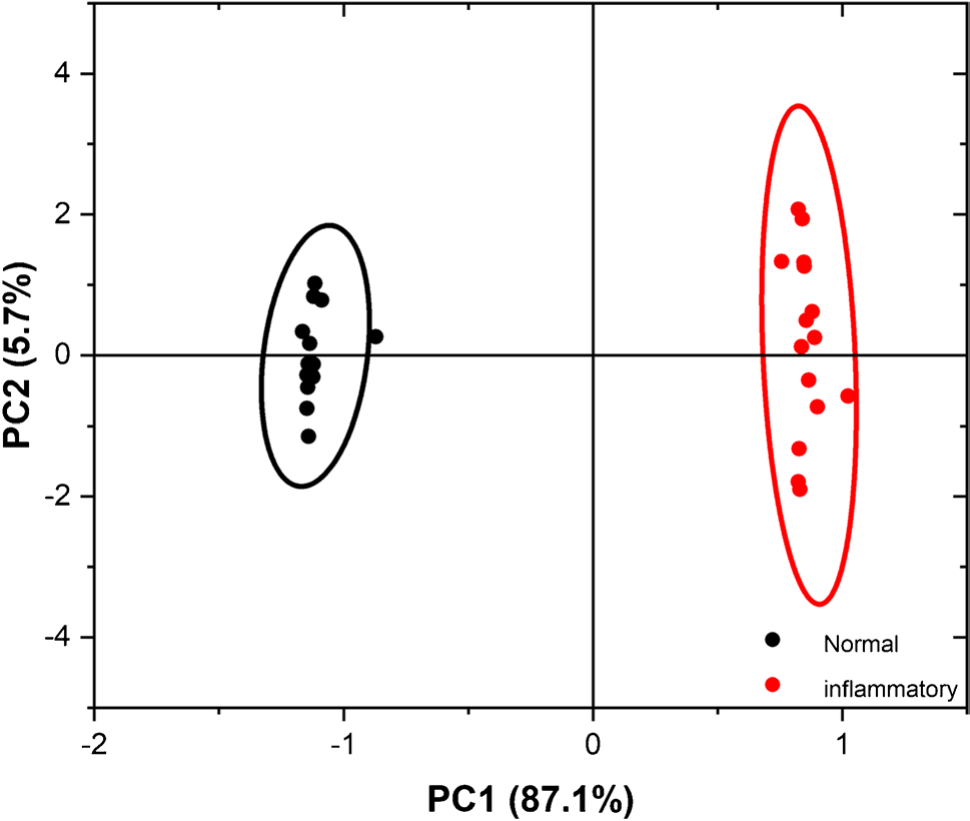
PCA score plot for the LIF spectra of normal and inflammatory blood serum samples

Twenty-five spectra from each sample type were utilized, encompassing the entire range of each spectrum (380–800 nm). The results are depicted in Fig. 6. Figure 6 shows that only two principal components were sufficient to distinctly differentiate between normal and inflammatory blood serum data points. Data from normal blood samples clustered on the negative side of PC1, whereas the majority of inflammatory blood samples’ data clustered on the positive side of PC1 on the plot. PC1 and PC2 represent 92.8% of the data variance, with PC1 accounting for 87.1% and PC2 for 5.7%. Hence, the PCA distinctly confirms a qualitative spectroscopic difference between normal and inflammatory samples, allowing for differentiation based on inflammation.

### Classification results based on graph theory

As discussed in the “Data analysis using graph theory” section, the obtained LIBS and LIF spectra are classified based on the graph theory approach.

#### Graph clustering of LIBS spectra

**Fig. 7:**
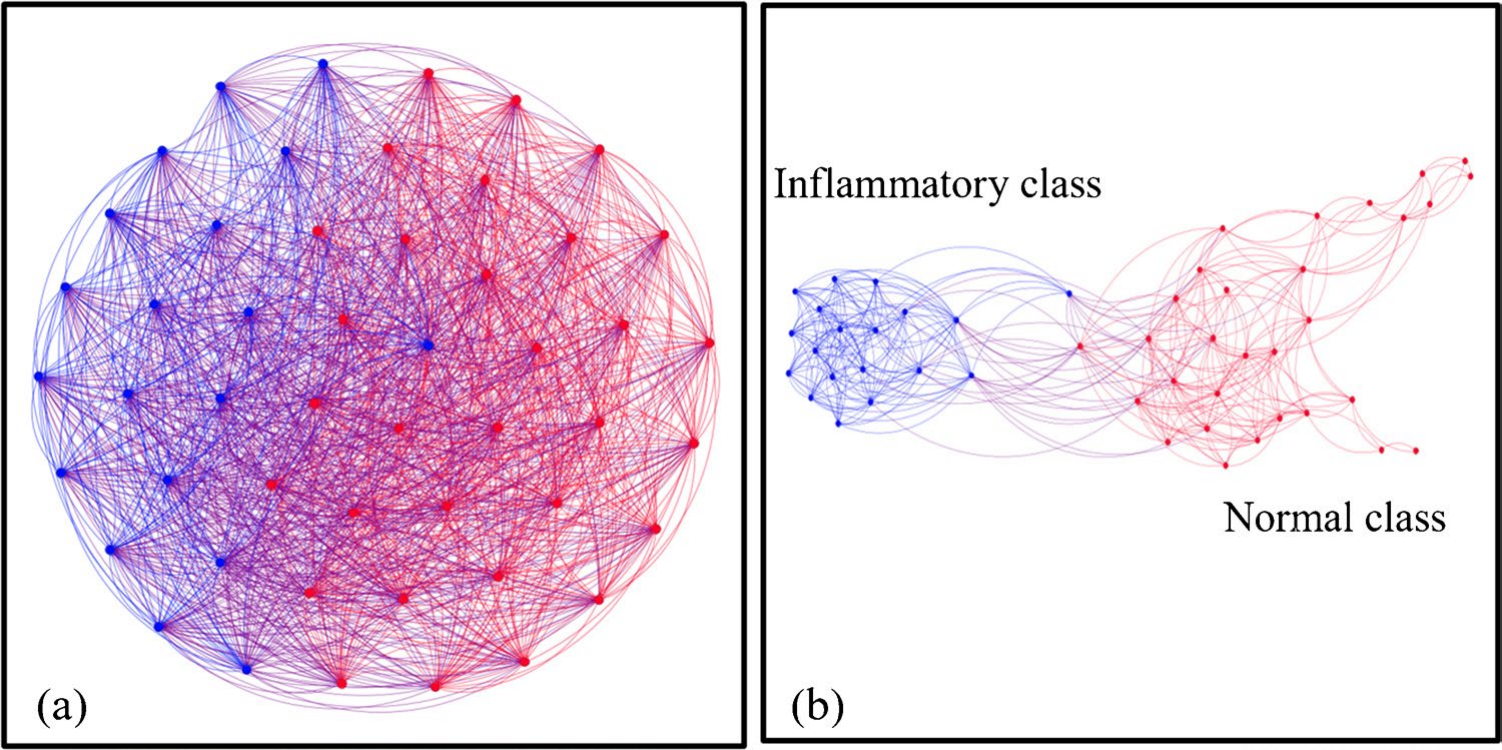
**a** Graphical representation of the graph obtained from the adjacency matrix, where the red edges connect the LIBS spectra corresponding to the normal class and the blue ones represent the inflammatory class, and **b** represents the separated classes after maximizing the modularity parameter by applying an edge weight filter of 0.9779.

The graph theory approach has been applied to classify the LIBS spectra of the samples under test. The obtained LIBS spectra have been used without manipulation. Figure 7 a shows the graphical representation of the used 50 spectra (i.e., 25 normal and 25 inflammatory). An edge weight filter with a value of 0.9779 has been applied for better separation between the two classes.

Hence, the modularity parameter has been maximized to 0*.*476 instead of 0.303 before applying the edge weight filter. It is worth noting that the increase of the modularity from 0.303 to 0.476 suggests that the clustering algorithm has identified communities with a more distinct and well-defined structure. The higher values of this parameter (nearly 1) generally indicate better-defined communities. A modulatory of 0.5 is often considered a good value, whereas a modulatory of less than 0.5 means the network does not exhibit any significant community structure.

As shown in Fig. 7 b, the two classes have been separated; however, the percentage of the normal class was 62%, and the inflammatory class was 38%, which is an inaccurate result. Additionally, there are some connections between the two classes. If the value of the edge weight filter is increased to greater than 0.9779, it yields four classes instead of two, which gives a poor classification result. The results show that the percentage of classification accuracy based on LIBS spectra was 76%, which was considered satisfactory.

#### Graph clustering of LIF spectra

**Fig. 8:**
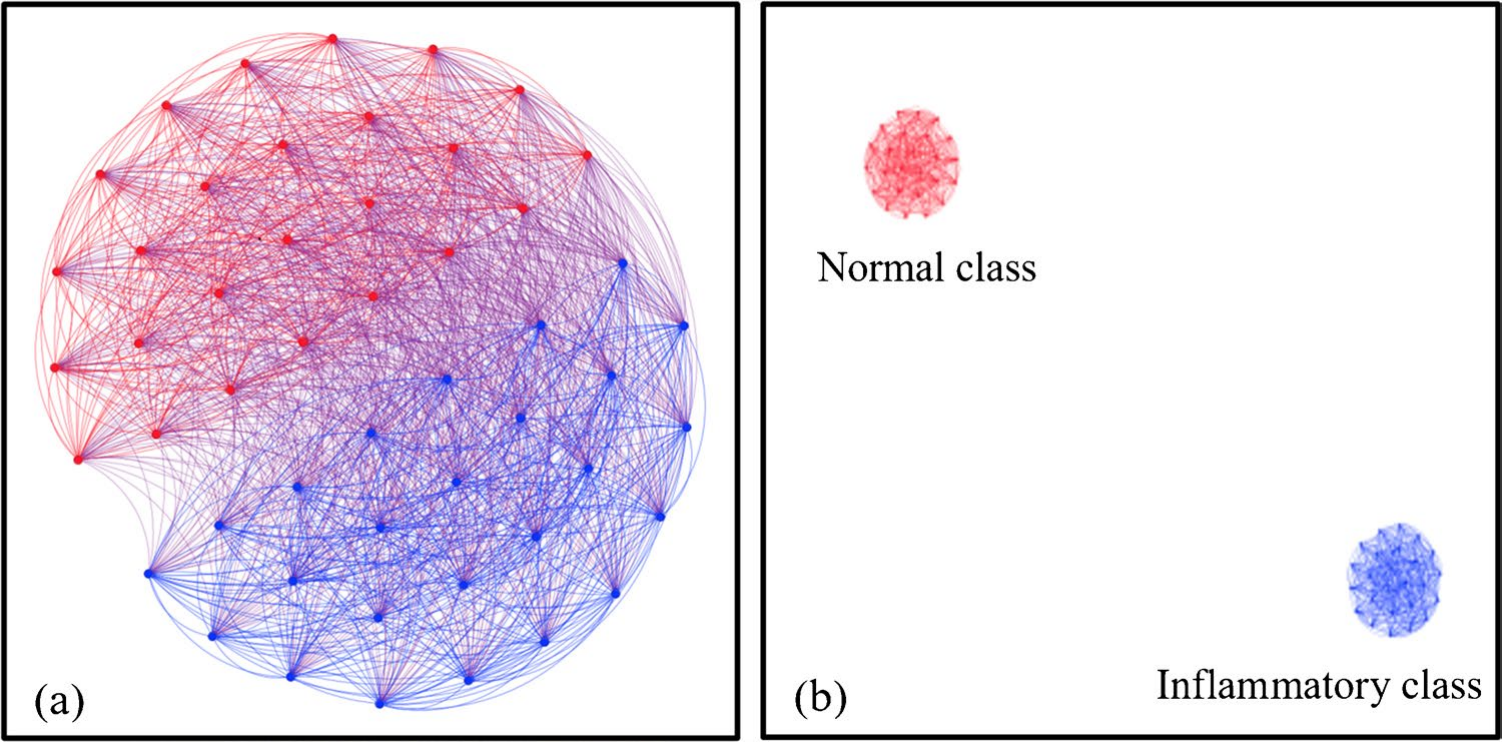
**A** Graphical representation of the graph obtained from the adjacency matrix, where the red edges connect the LIF spectra corresponding to the normal class, the blue ones represent the inflammatory class, and **b** represents the separated classes after maximizing the modularity parameter by applying an edge weight filter of 0.854

Significant discrimination between the two groups, normal and inflammatory, could be effectively obtained using the graph clustering approach, as depicted in Fig. 8. This method is an intrinsically unsupervised classifier (i.e., the samples are automatically grouped based on their similarity via a graph algorithm). The open-source “Gephi software” [46] has been used to provide a graphical representation of the graph described by the correlation matrix (Eq. 1). The modularity of the untreated adjacency matrix is relatively low (0.057), and its graphical representation is depicted in Fig. 8 a. The results showed that the two groups are linked together. Applying an edge weight filter with a weight of 0.854 allows better separation between the two groups, as shown in Fig. 8 b. In such a case, the modularity parameter is maximized to be 0.5. The results reveal that the graph theory approach classified the two groups with an accuracy of 100%.

It is worth mentioning that while the LIF measurements alone may provide clear differentiation in the current study, the addition of LIBS and the utilization of the graph clustering technique could offer complementary information, enhance robustness, facilitate exploratory analysis, and lay the groundwork for future extensions or applications of this study. LIF often provides superior results because it focuses on the emission from specific molecular bands, simplifying spectral analysis. In contrast, the LIBS spectrum is complex due to numerous emission spectral lines from multiple elements, which can be resolved or unresolved, making spectral interpretation more challenging, depending on the instrument’s resolving power of closely spaced spectral lines. In this work, the two techniques, LIBS as an elemental spectrochemical analysis technique and LIF as a molecular one validate each other’s outcomes. In addition, we are conducting a detailed univariate analysis for these samples in another work under publication that correlates: (i) in LIBS, a specific relevant analyte against CRP, and (ii) in LIF, a significant spectral integral against CRP.

## Conclusions

In the current study, two spectrochemical analytical techniques, LIBS and LIF, were utilized to discriminate between normal and inflammatory blood serum samples. In the LIBS technique, the obtained spectra showed a pronounced difference in calcium levels between the two classes in the wavelength range from 315 to 440 nm. The higher levels of calcium in blood serum are associated with inflammatory conditions. In the LIF technique, exciting blood serum samples with a laser with a wavelength of 405 nm results in a distinctive emission peak being observed at a wavelength of 518 nm. Its fluorescence intensity in a normal blood serum sample was 5.3 times higher than in the inflammatory samples. This can be attributed to the difference in the concentration of specific fluorophores between the two groups of samples. Two approaches were employed to classify and cluster the two classes: PCA and graph theory. The PCA effectively classified and discriminated between normal and inflammatory blood serum samples with a data variance of 85*.*4% and 92*.*8% based on LIBS and LIF spectra. The unsupervised graph theory approach also grouped the obtained spectra from LIBS and LIF with 76% and 100% classification accuracy, respectively. In conclusion, unlike conventional laboratory clinical analyzers, the techniques offered in the current work represent a simple, rapid, non-destructive, label-free, and cost-effective approach to inflammation detection.

## Data Availability

The data generated and analyzed during the current study are available from the corresponding author upon request
